# Data on in vivo phenotypes of GFRα1-positive spermatogonia stimulated by interstitial GDNF signals in mouse testes

**DOI:** 10.1016/j.dib.2016.07.055

**Published:** 2016-08-03

**Authors:** Aya Uchida, Yoshiakira Kanai

**Affiliations:** Department of Veterinary Anatomy, The University of Tokyo, Yayoi, Tokyo 113-8657, Japan

**Keywords:** Testis, Spermatogonial stem cell, Spermatogenesis, GDNF, Bead technology, Transplantation

## Abstract

This article contains the data related to the research article “in vivo dynamics of GFRα1-positive spermatogonia stimulated by GDNF signals using a bead transplantation assay” (Uchida et al., 2016) [1]. A novel transplantation assay of growth factor-soaked beads into the mammalian testicular interstitium was developed, in order to examine the effects of various soluble factors on in vivo dynamics of the spermatogonia including spermatogonial stem cells (SSC). Here we provide the image data of GFRα1-positive stem/progenitor spermatogonia in mouse seminiferous tubules near the beads soaked in GDNF (glial cell-derived neurotrophic factor), one of the SSC niche factors. The data provide various phenotypes of GFRα1-positive spermatogonia induced by bead-derived GDNF signals, which are useful to understand the active state of GFRα1-positive stem/progenitor spermatogonia in vivo.

**Specifications Table**

**Table:** 

Subject area	*Biology*
More specific subject area	*Spermatogenesis, Stem cell biology*
Type of data	*Image*
How data was acquired	*Images taken by an Olympus fluorescence microscope (BX51N-34-FL2).*
Data format	*Raw*
Experimental factors	*Beads were soaked into the solution of GDNF (0.1 mg/ml) for 1 h at room temperature.*
Experimental features	*GDNF-soaked beads were labeled with DiI fluorescent dye, and then transplanted into testis interstitium via vitrified micro-capillary. After days 3–5 post-transplantation, GFR*α*1-positive spermatogonia in the seminiferous tubules near DiI-labeled sites were analyzed by whole-mount immunofluorescence.*
Data source location	*The University of Tokyo, Tokyo, Japan*
Data accessibility	*Data is within this article*

**Value of the data**

• The image data provide various phenotypes of the GFRα1-positive stem/progenitor spermatogonia near GDNF-soaked beads which were transplanted into the interstitium of mouse testes in vivo.
• These image data are useful for the estimation of the active state of GFRα1-positive spermatogonia by comparing their phenotypic similarities to those in other testes under various conditions.
• These data allow researchers to elucidate the effects of various growth factors in mammalian spermatogenesis in vivo.
• This bead transplantation technique will be helpful for the preservation and renewal in sub- and infertile males of various mammal species.

## 1 Data

**Fig. 1:**
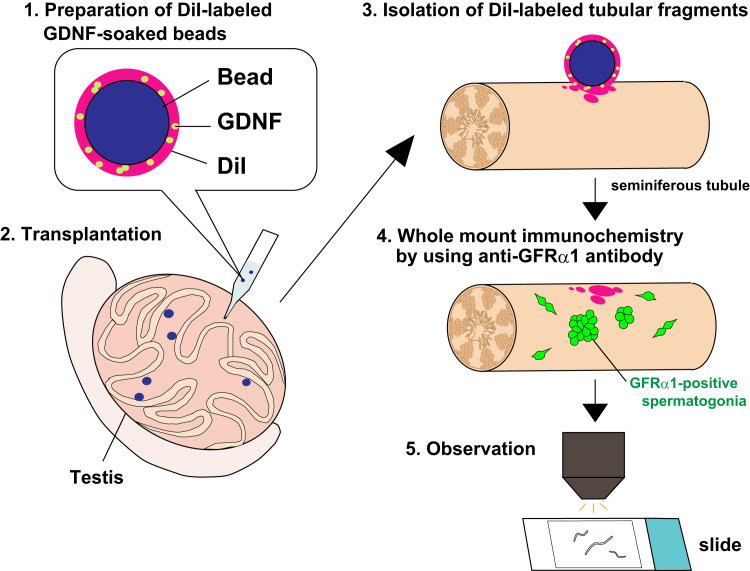
Schematic illustration of the in vivo transplantation of DiI-labeled/GDNF-soaked beads into mouse testicular interstitium. 1. Micro-beads (approximately 100 μm in diameter) were soaked in solutions of recombinant GDNF and DiI. 2. Beads were transplanted into mouse testicular interstitium via vitrified micro-capillary with certain intervals. 3. The beads-transplanted testes were extracted at days 1, 3, 5 post-transplantation, and then DiI-labeled tubular fragments were isolated for further analyzes. 4. To visualize undifferentiated spermatogonia, whole mount anti-GFRα1 immunostaining was conducted to the isolated tubular fragments. 5. The immunostained samples were observed by microscopy.

**Fig. 2:**
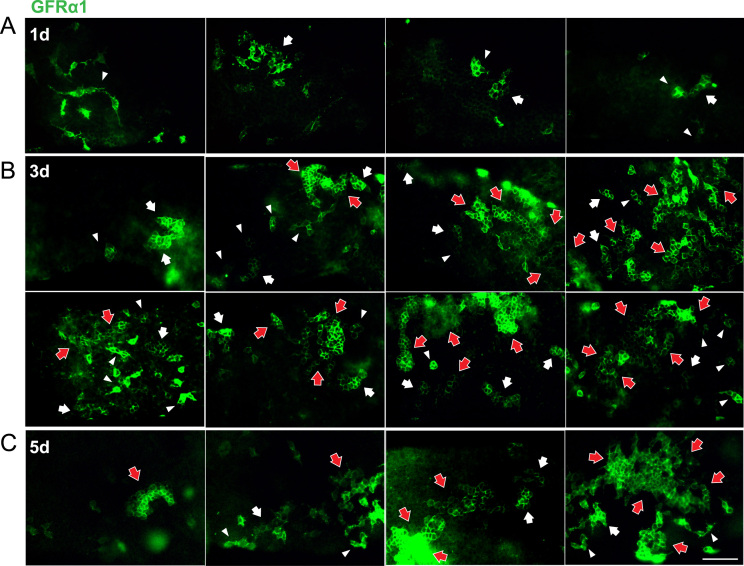
Phenotypes of GFRα1-positive spermatogonia (green) in the seminiferous tubules close to GDNF-soaked beads of days 1 (A), 3 (B) and 5 (C) post-transplants. In A–C, the figures are arranged in ascending order of the number of large GFRα1-positive cells aggregate, from left to right. Whole-mount anti-GFRα1 staining (green) of GDNF -soaked bead transplants at days 1, 3, 5 post-transplantation. White arrowhead, white arrow or red arrow indicates an aggregate with 4–7, 8–31, and more than 32 GFRα1-positive cells, respectively. Bar, 100 μm.

A novel bead transplantation assay into mouse testicular interstitium (Fig. 1) [1] provides the immunofluorescence image data showing the various phenotypes of the GFRα1-positive stem/progenitor spermatogonia near the GDNF-soaked beads at days 1, 3 and 5 post-transplantation (Fig. 2).

## 2 Experimental design, materials and methods

### 2.1 Animals

Wild-type male mice (8-week-old, ICR strain; SLC Inc.) were used. All animal experiments were performed in strict accordance with the Guidelines for Animal Use and Experimentation at the University of Tokyo (Approval IDs: P13-762, P16-083).

### 2.2 Bead preparation and transplantation

Affi-Gel blue beads (approximately 100 μm in diameter; Bio Rad) were soaked in a solution of recombinant GDNF (0.1 mg/ml; Calbiochem) for 1 h at room temperature. To mark the tubular wall adjacent to the transplanted beads, the beads were immersed in DiI (0.83 mg/ml; Thermo Fisher Scientific) solution for 15 min.

For transplantation, the adult testes were gently extracted from the abdominal cavity under anesthesia, and then the GDNF- or BSA (negative control)-soaked beads were transplanted into the testicular interstitium (approximately eight beads [one or two beads per site] were separated by appropriate intervals) via vitrified micro-capillary under a dissecting microscope.

### 2.3 Immunohistochemistry

For whole-mount immunostaining, seminiferous tubule fragments around the transplanted beads were isolated, fixed in 4% PFA-PBS for 8 h at 4 °C, and washed with PBST, as described previously [2]. The fragments were stained with anti-GFRα1 (1:100 dilution; R&D Systems) antibody, in combination with the secondary antibodies conjugated with Alexa-488. The stained samples were photographed by using an Olympus fluorescence microscope (BX51N-34-FL2).
